# Adherence to postresection colorectal cancer surveillance at National Cancer Institute‐designated Comprehensive Cancer Centers

**DOI:** 10.1002/cam4.1678

**Published:** 2018-10-18

**Authors:** Sonia S. Kupfer, Sam Lubner, Emmanuel Coronel, Perry J. Pickhardt, Matthew Tipping, Peter Graffy, Eileen Keenan, Eric Ross, Tianyu Li, David S. Weinberg

**Affiliations:** ^1^ Section of Gastroenterology, Hepatology and Nutrition University of Chicago Chicago Illinois; ^2^ University of Wisconsin School of Medicine & Public Health Madison Wisconsin; ^3^ Fox Chase Cancer Center Philadelphia Pennsylvania

**Keywords:** colorectal cancer, surveillance, survivorship

## Abstract

Guidelines recommend surveillance after resection of colorectal cancer (CRC), but rates of adherence to surveillance are variable and have not been studied at National Cancer Institute (NCI)‐designated Comprehensive Cancer Centers. The aim of this study was to determine rates of adherence to standard postresection CRC surveillance recommendations including physician visits, carcinoembryonic antigen (CEA), computed tomography (CT), and colonoscopy after CRC resection at three NCI‐designated centers.

Data on patients with resected CRC from 2010 to 2017 were reviewed. Adherence to physician visits was defined as having at least two visits within 14 months after surgical resection. CEA adherence was defined as having at least four CEA levels drawn within 14 months. CT and colonoscopy adherence were defined as completing each between 10 and 14 months from surgical resection. Chi‐square test and logistic regression analyses were performed for overall adherence and adherence to individual components.

A total of 241 CRC patients were included. Overall adherence was 23%. While adherence to physician visits was over 98%, adherence to CEA levels, CT, and colonoscopy were each less than 50%. Center was an independent predictor of adherence to CEA, CT, and/or colonoscopy. Stage III disease predicted CT adherence, while distance traveled of 40 miles or less predicted colonoscopy adherence.

Overall adherence to postresection CRC guideline‐recommended care is low at NCI‐designated centers. Adherence rates to surveillance vary by center, stage, and distance traveled for care. Understanding factors associated with adherence is critical to ensure CRC patients benefit from postresection surveillance.

## 1 INTRODUCTION

Colorectal cancer (CRC) is the third most common cancer in the United States (US) and the second most common cause of cancer‐related mortality in men and women.^1^ Despite curative surgical resection and medical treatment in nonmetastatic CRC patients (stage II and III), about 30% of patients will have disease recurrence.^2^ The goal of postoperative surveillance was to identify patients whose cancers recur to offer therapies with the potential to improve survival. While the benefit of intensive surveillance is debated, some, but not all, randomized controlled trials^3^, ^4^, ^5^, ^6^, ^7^, ^8^, ^9^, ^10^, ^11^, ^12^, ^13^, ^14^ and meta‐analyses^15^, ^16^, ^17^, ^18^, ^19^, ^20^ support increased rates of curative‐intent surgery and/or modest survival benefit. For example, a large meta‐analysis of 4,055 patients undergoing intensive surveillance strategies demonstrated increased detection of disease recurrence and corresponding increased rate of curative surgery and increased survival.^19^ A prospective randomized study from the United Kingdom confirmed that either carcinoembryonic antigen (CEA) or computed tomography (CT) increased the odds of having a curative‐intent surgery, although there was no overall survival benefit of this strategy.^14^ Importantly, CRC surveillance has been found to be cost‐effective.^21^

Several organizations have published guidelines for postoperative surveillance of resected CRC. While there are some differences among the guidelines, the American Society of Clinical Oncology (ASCO), National Comprehensive Cancer Network (NCCN), and the American Society of Colon and Rectal Surgeons (ASCRS),^22^, ^23^, ^24^ all recommend physician visits with a comprehensive history and physical examination and CEA measurement every 3‐6 months as well as chest, abdominal and pelvis CT and colonoscopy 12 months after surgery (assuming a clearing colonoscopy was performed preoperatively). Colonoscopy surveillance is recommended for stage I‐III, whereas CEA and imaging apply to stage II and III patients as well as stage I patients with high‐risk features per ASCRS.^24^ Adherence to these guidelines is variable with rates between 12% and 87% based on a systematic review.^25^ This wide range likely reflects differences in definitions of adherence based on changing guidelines as well as differences in patient populations and practice settings.

No recent studies of adherence to guidelines for patients with resected CRC have been published, and no study has included US National Cancer Institute (NCI)‐designated Comprehensive Cancer Centers where adherence to guidelines has been shown to be higher than non‐NCI‐designated centers.^26^ However, in the context of enrollment for a prospective observational clinical trial comparing postresection optical colonoscopy to CT colonography for CRC surveillance at NCI‐designated Comprehensive Cancer Centers (NCT02143115), we observed that receipt of postresection surveillance appeared to vary widely. Therefore, we sought to determine surveillance rates and associated factors at three participating NCI‐designated centers.

## 2 METHODS

### 2.1 Data collection

This was a retrospective cohort study performed at three NCI‐designated Comprehensive Cancer Centers in the US. Two centers were located in the Midwestern US and one in the Northeastern US. All three participating institutions received Institutional Review Board approval. Data on adult patients (over 18 years of age) with resected, nonmetastatic CRC (stages I‐III) at diagnosis were collected retrospectively from medical records. Patient demographics, physician visits, CEA levels, CT examinations of the chest, abdomen, and pelvis for the indication of surveillance and colonoscopies were reviewed and recorded. In cases where a test (CEA, CT and/or colonoscopy) was not performed at the NCI center, records from outside hospitals where the test was performed were reviewed. The distance traveled by patients to their center of care was calculated based on their zip code. The study period was 2010‐2017, and the observation period was 14 months from the date of initial CRC surgical resection. No obstructing cancers were included.

### 2.2 Definitions of adherence to surveillance

Physician visit adherence was defined as ≥2 visits to a physician (medical oncologist, surgeon, etc.) within the study period (up to 14 months). Adherence to CEA was defined as at least four levels drawn in the 14‐month time period after surgical resection. Overutilization of CEA was defined as receiving more than four CEA measurements in this time frame. CT and colonoscopy adherence were defined as completing each test between 10 and 14 months from surgical resection. This range was selected to account for scheduling. Overall adherence was defined as completing all four components (physician visit, CEA, CT, and colonoscopy) within the defined time period. Patients with less than 14 months of follow‐up after initial resection and/or those with stage IV disease were excluded from the study.

### 2.3 Statistical analyses

Chi‐square tests were used to assess bivariate associations between demographic or clinical factors such as age group, race, gender, stage, center, and distance travelled to center for care (≤40 miles vs >40 miles) with overall adherence and individual components of surveillance. Multivariable logistic regressions were used to model overall adherence and its components. All tests were two‐sided with a 5% type I error. Analyses were conducted using SAS 9.4 (Cary, NC).

## 3 RESULTS

In total, 241 patients were included. Patient characteristics are shown in Table 1. Median age was 64 years with a predominance of patients over age 50. There were more women than men, and the majority was white. More patients with stage II and III disease were included compared with stage I. There were more patients from center B compared with the other two centers. About 83% of the patients traveled 40 miles or less from home for care. In total, 76.8% of patients had a documented medical oncology visit, and 77.2% had a visit in surgery clinic. The majority of patients (57.7%) were seen in both medical oncology and surgery clinics.

**Table 1:** Patient demographics, n = 241

Mean age at diagnosis (SD)	64.1 (14.6)
Age at diagnosis (%)	
<50 y	38 (15.8)
50‐64 y	84 (34.8)
65‐75 y	50 (20.8)
≥75 y	69 (28.6)
Sex (%)	
Female	141 (58.5)
Male	100 (41.5)
Race (%)	
White	193 (80.1)
Black	31 (12.9)
Other	17 (7.0)
Location (%)	
Colon	185 (76.8)
Rectum	56 (23.2)
Stage (%)	
I	54 (22.4)
II	78 (32.4)
III	109 (45.2)
Center (%)	
A	78 (32.4)
B	92 (38.2)
C	71 (29.4)
Distance to Center (%)	
>40 miles	39 (16.5%)
≤40 miles	198 (83.5%)

The rate of adherence to all surveillance modalities within the study period was 22.8%. The majority (98.3%) were adherent to physician visits. Adherence to CEA, CT and colonoscopy were each under 50%. For CEA measurements, one‐third of patients had more CEA levels drawn in the study period (“over‐use”). CEA use was significantly different by stage, center, and distance travelled (Table 2). Specifically, there were increased rates of CEA over‐use in stage II & III, center C and among those who travel less than 40 miles to their center.

**Table 2:** Bivariate analysis for overall adherence and adherence to individual components

	All n (%)	CEA n (%)	CT n (%)	Colonoscopy n (%)
Age				
<50 y	11 (29.0)	18 (47.4)	19 (50.0)	19 (50.0)
50‐64 y	21 (25.0)	45 (53.6)	43 (51.2)	35 (41.2)
65‐74 y	12 (24.0)	22 (44.0)	22 (44.0)	16 (32.0)
≥75 y	11 (15.9)	27 (39.1)	25 (36.2)	30 (43.5)
Sex				
Female	31 (22.0)	67 (47.5)	69 (48.9)	60 (42.6)
Male	24 (24.0)	45 (45.0)	40 (40.0)	40 (40.0)
Race				
White	44 (22.8)	88 (45.6)	87 (45.1)	80 (41.5)
Black	4 (12.9)	13 (42.0)	11 (35.5)	12 (38.7)
Other	7 (41.2)	11 (64.7)	11 (64.7)	8 (47.1)
Stage				
I	7 (13.0)	20 (37.0)*	16 (29.6)***	23 (42.6)
II	20 (25.6)	35 (44.9)	33 (42.3)	35 (44.9)
III	28 (25.7)	57 (52.3)	60 (55.1)	42 (38.5)
Center				
A	6 (7.7)*	30 (38.5)*	26 (33.3)***	25 (32.1)*
B	38 (41.3)	55 (59.8)	54 (58.7)	55 (59.8)
C	11 (15.5)	27 (38.0)	29 (40.9)	20 (28.2)
Distance				
>40 miles	4 (10.3)	15 (38.5)	12 (30.8)	7 (18.0)
≤40 miles	51 (25.8)**	95 (48.0)***	97 (50.0)**	92 (46.5)*

*P < 0.001; **P < 0.05; ***P < 0.01.

On univariate analysis (Table 3), there were significant differences in overall adherence by center and distance travelled. Center B had a significantly higher rate of overall adherent patients (41.3%) compared to centers A and C (7.7% and 15.5%, respectively). Patients who traveled 40 miles or less were more adherent overall (25.8%) than those who traveled more than 40 miles (10.3%). There was a trend toward greater overall adherence by stage but this did not reach statistical significance. When considering each modality individually, there were significant differences for CEA, CT, and colonoscopy by center and distance traveled. Increasing rates of adherence to CEA and CT but not colonoscopy were noted by stage.

**Table 3:** Bivariate analysis of CEA adherence and over‐use. CEA over‐use defined as more than four CEA measurements in the 14‐month study period

	Not adherent n (%)	Adherent n (%)	Over‐use n (%)	*P*‐value
Age				0.13
<50 y	6 (15.8)	18 (47.4)	14 (36.8)	
50‐64 y	13 (15.5)	45 (53.6)	26 (30.9)	
65‐74 y	13 (26.0)	22 (44.0)	15 (30.0)	
≥75 y	24 (34.8)	27 (39.1)	18 (26.1)	
Sex				0.88
Female	33 (23.4)	67 (47.5)	41 (29.1)	
Male	23 (23.0)	45 (45.0)	32 (32.0)	
Race				0.29
White	49 (25.4)	88 (45.6)	56 (29.0)	
Black	6 (19.3)	13 (42.0)	12 (38.7)	
Other	1 (5.9)	11 (64.7)	5 (29.4)	
Stage				**<0.001**
I	28 (51.8)	20 (37.0)	6 (11.1)	
II	14 (18.0)	35 (44.9)	29 (37.2)	
III	14 (12.8)	57 (52.3)	38 (34.9)	
Center				**<0.001**
A	37 (47.4)	30 (38.5)	11 (14.1)	
B	9 (9.8)	55 (59.8)	28 (30.4)	
C	10 (14.1)	27 (38.0)	34 (47.9)	
Distance				**0.0006**
>40 miles	18 (46.2)	15 (38.5)	6 (15.4)	
≤40 miles	37 (18.7)	95 (48.0)	66 (33.3)	

On multivariate analysis (Table 4), odds of overall adherence were significantly increased in center B compared to centers A and C. None of the other factors in the model (age, gender, race, stage, or distance travelled) were significant independent predictors of overall adherence. For CEA, CT, and colonoscopy, center B was a positive predictor of adherence to CT and colonoscopy, while center C was a negative predictor of adherence to CEA. Stage III disease was a significant predictor of adherence to CT, while traveling 40 miles or less was a significant positive predictor of adherence to colonoscopy. For univariate and multivariate analyses, physician visits were not included due to high rates of adherence that limited these analyses.

**Table 4:** Multivariate analysis. Shown are odds ratios (95% two‐sided confidence intervals)

	All	CEA	CT	Colonoscopy
Age				
<65 y	Ref	Ref	Ref	Ref
≥65 y	1.03 (0.51‐2.06)	0.73 (0.43‐1.22)	0.91 (0.51‐1.62)	0.84 (0.46‐1.52)
Gender				
Male	Ref	Ref	Ref	Ref
Female	0.90 (0.46‐1.76)	1.02 (0.62‐1.67)	1.45 (0.83‐2.52)	1.15 (0.65‐2.03)
Race				
Black	Ref	Ref	Ref	Ref
White	2.57(0.80‐8.20)	1.11 (0.51‐2.42)	1.88 (0.80‐4.43)	1.30(0.52‐3.22)
Other	4.51 (1.0‐20.67)	1.88 (0.57‐6.20)	2.91 (0.79‐10.72)	1.45 (0.38‐5.61)
Stage				
I	Ref	Ref	Ref	Ref
II	1.47 (0.52‐4.20)	0.80 (0.40‐1.60)	1.40 (0.63‐3.09)	0.77 (0.35‐1.72)
III	1.35 (0.49‐3.75)	0.88 (0.44‐1.74)	**2.20 (1.02‐4.76)** a	0.50 (0.23‐1.12)
Center				
A	Ref	Ref	Ref	Ref
B	**7.59 (2.85‐20.20)** a	1.25 (0.67‐2.32)	**2.33 (1.18‐4.62)** a	**3.09 (1.52‐6.28)** a
C	2.25 (0.73‐6.91)	**0.46 (0.23‐0.92)** a	1.32 (0.62‐2.84)	0.80 (0.36‐1.80)
Distance				
>40 miles	Ref	Ref	Ref	Ref
≤40 miles	2.40 (0.76‐7.62)	0.95 (0.48‐1.86)	1.85 (0.85‐4.05)	**4.05 (1.63‐10.08)** a

a *P*‐value < 0.05; Ref, reference.

## 4 DISCUSSION

Reported rates of adherence to surveillance by patients with resected CRC are variable,^16^, ^25^ and clinical surveillance practice patterns have not been measured recently. It is important to understand factors associated with adherence to postresection CRC in order to ensure patients benefit in terms of curative‐intent surgery and survival. In this multicenter study at NCI‐designated Comprehensive Cancer Centers, patients with resected CRC had low overall rates of adherence to published surveillance guidelines. While adherence to physician visits was more than 90%, rates of adherence to CEA, CT, and colonoscopy were each individually suboptimal under 50%. Of all the surveillance components, adherence to colonoscopy was lowest at 41.5%. The strongest independent predictor of overall adherence was the center where patients received care. Stage III disease was an independent predictor of receipt of CT, and travel of 40 miles or less was an independent predictor of receipt of surveillance colonoscopy.

The range of previously reported rates of overall adherence to CRC surveillance is 12%‐87%.^25^ It should be noted that each study included different definitions of adherence (reflecting variability of guideline recommendations over time), different observation time periods and unique patient populations that likely explains the wide range of estimates. The current study is unique in that none of the previous studies included NCI‐designated Comprehensive Cancer Centers where CRC surgery and chemotherapy‐related outcomes have been reported to be higher compared to nondesignated centers.^27^, ^28^ The present study did not collect outcome data limiting the ability to determine how surveillance impacted specific outcomes in patients at these centers.

The strongest independent factor associated with overall adherence was the center where care was provided. This is in line with previous studies that have reported differences in adherence to post‐CRC resection surveillance across different geographic and health systems.^29^, ^30^, ^31^ Regional differences could reflect different practice patterns or delivery systems, though all sites in the current study were NCI‐designated Comprehensive Cancer Centers in academic hospitals albeit in different geographic areas. Factors that influence adherence such as use of a survivorship plan could also contribute to differences between centers, but this information was not collected in this study. Another factor that has been shown to be associated with differences in adherence rates for surgery among NCI‐designated and nondesignated centers is the strength of evidence of a particular guideline.^32^ While there is some debate about the value of post‐CRC resection surveillance, the surveillance strategy measured in the present study has broad consensus across multiple guidelines and is therefore less likely to explain variation by center.

Previous studies have reported that increasing age, nonwhite race, increased comorbidities and pre‐operative complications, contact with a physician as well as receipt of adjuvant chemotherapy are significantly associated with adherence to CRC surveillance.^25^ Based on multivariate analysis in the present study, patient age, race, and gender did not account for differences in adherence rates. It is possible that factors such as comorbidities, insurance type, education, or income could contribute to adherence but were not measured in this study.

When considering each component of recommended surveillance individually, adherence to physician visits was uniformly high. Measurement of CEA, on the other hand, was only 45% across all centers and the only independent predictor of adherence to CEA was center. In this study, we were able to assess the impact of overutilization for CEA and found that a third of patients received more frequent CEA measurements over the time period than were recommended. Overutilization differed by stage and center with one center having higher rates of over‐use compared to appropriate use. Overutilization can lead to increased health care costs among other negative effects and should be addressed similarly to underutilization.^33^

Rates of CT and colonoscopy adherence between 10 and 14 months after surgical resection were each below 50% in the current study. Previous studies have demonstrated similar suboptimal rates of adherence for CT and colonoscopy.^34^ For both CT and colonoscopy, center was an independent predictor of adherence. As was observed for overall adherence, differences between centers could reflect individual or regional practice patterns. Distance traveled of 40 miles of less was an independent predictor of colonoscopy adherence. This is perhaps not surprising given that a bowel purge is required and could be a barrier for patients living farther away from the tertiary care center. The effect of distance from the medical center has not been previously studied for CRC surveillance but has been reported to predict adherence to recommended treatment of gynecological cancers in the US including at NCI‐designated centers.^35^, ^36^ In contrast, distance traveled did not affect receipt of chemotherapy for CRC patients in Australia.^37^ Stage III disease was an independent predictor of CT adherence that could be explained by increased risk of recurrent disease. A limitation of the current study is that rates of CT scans and colonoscopy before 10 months were not available but could partially explain low rates of adherence during the predefined adherence window of 10‐14 months after resection, although earlier examinations are unlikely to be as useful regarding curative resection and survival. Additional factors that have previously been associated with adherence to imaging and colonoscopy such as patient demographics (age, gender, socioeconomic status, insurance) were not significant predictors or were not measured in the current study.

Strengths of the current study include inclusion of NCI‐designated Comprehensive Cancer Centers in various geographic regions. This population has not been previously studied in the context of adherence to CRC surveillance. Limitations include short follow‐up and lack of data regarding early use of CT and colonoscopy to assess overutilization or early use of surveillance modalities. The modest sample size also limited power for assessment of overall adherence given low rates of overall adherence. We were not able to stratify stage I patients according to specific high‐risk criteria as suggested by ASCRS guidelines for surveillance using CEA and imaging.^24^ Finally, adherence could be due to lack of physician recommendation and/or patient noncompliance; however, it was not possible to dissect reasons for lack of adherence in this retrospective study.

In summary, this study demonstrates an unacceptably low rate of overall adherence to CRC surveillance guideline recommendations at NCI‐designated Comprehensive Cancer Centers. The center where patients receive their care was an important predictor of adherence and highlights the need for quality improvement in centers that perform less well. Efforts to improve adherence in these practice settings are urgently needed to ensure quality care for CRC survivors.

## CONFLICT OF INTERESTS

None of the authors have conflict of interests to report.

## AUTHOR CONTRIBUTIONS

All authors participated in the research and preparation of the manuscript.
